# Silver nanoparticles coated with metabolites of *Pseudomonas* sp. N5.12 inhibit bacterial pathogens and fungal phytopathogens

**DOI:** 10.1038/s41598-024-84503-z

**Published:** 2025-01-09

**Authors:** Svitlana Plokhovska, Ana García-Villaraco, Jose Antonio Lucas, Francisco Javier Gutierrez-Mañero, Beatriz Ramos-Solano

**Affiliations:** 1 Faculty of Pharmacy, Universidad San Pablo-CEU Universities, 28668-Boadilla del Monte, Madrid, Spain; 2 Institute of Food Biotechnology and Genomics, NAS of Ukraine, Baidy-Vyshnevetskoho Str. 2а, Kyiv, Ukraine

**Keywords:** Silver nanoparticles (AgNP), Biological synthesis, Plant growth promoting bacteria (PGPB), Antibacterial and antifungal activity, Biotechnology, Microbiology, Plant sciences, Nanoscience and technology

## Abstract

The synthesis of nanomaterials from PGPB is an exciting approach and it’s often used in agriculture as nano-fertilizers and nano-pesticides. The present study reports a new approach to biosynthesis of silver nanoparticles (AgNP), using bacterial metabolites as agents to reduce Ag^+^, which will remain as coating agents able to prevent microbial growth. Silver NP were biosynthesized using the bacterial metabolites produced by the beneficial strain *Pseudomonas* sp. N5.12. Optimization of physicochemical parameters (temperature, pH, and AgNO_3_ concentration) for the synthesis of AgNP was carried out. In each condition, success on AgNP synthesis was determined by UV–Visible spectra showing peaks between 400 and 450 nm. TEM analysis showed that the AgNP are spherical in shape with an average particle size ranging from 13.75 ± 0.47 nm to 20.71 ± 0.43 nm, covered with a unique organic matter corona of bacterial metabolites. The best parameters for AgNP biosynthesis by *Pseudomonas* sp. N5.12 occurred with 24 h bacterial metabolites, temperature of 37 °C, pH 9 and a ratio of 2:4 (v: v; bacterial supernatant: 1 mM AgNO_3_). The biosynthesized AgNP inhibited growth of human pathogenic bacteria better than equivalent AgNO_3_ concentration. Growth of bacterial and fungal phytopathogens was also inhibited with striking effects on *Alternaria* sp. (74% inhibition) and *Stemphylium* sp. (52% inhibition), appearing as promising tools to biocontrol fungal diseases in agriculture.

**Supplementary Information:**

The online version contains supplementary material available at 10.1038/s41598-024-84503-z.

## Introduction

Nanotechnology appears as an exciting field of research dealing with an array of nanomaterials of different shape, size and compositions with applications in several fields, varying from medicine to agriculture^1^. Nanoparticles (NP) are defined as ultrafine organic and inorganic materials, between 1 and 100 nm, that share the presence of a reduced metal. Among the different metals like iron, zinc, gold, silver, selenium, and nickel that are being explored for synthesis of NPs, silver has the advantage of chemical inertness, easy reduction, electrical conductivity, biosensor capacity and wide implementation as antimicrobial agent formulated as silver nanoparticles (AgNP). Hence, AgNP have been established in biomedical and biological applications as one of the promising nanoparticles due to their unique physical and chemical properties^2^. AgNP have antibacterial properties and are well known as an effective disinfectant against a wide range of microorganisms and can also treat bacterial infections through longer exposure times due to their good stability^3^. A large number of Gram-positive and Gram-negative bacteria, fungi and viruses are sensitive to AgNP^4,5^. The proposed mechanism of action^6^ of AgNP is based on the ability of NP to release of Ag^+^ions; these ions interact with membranes altering cell homeostasis, penetrate cells where interactions with biomolecules occur directly or as a result to concomitant reactive oxygen species increase^7^. However, their ability to partly destroy pathogen membrane has recently been reported^8^. So far, these mechanisms refer to non-biologically synthesised AgNP.

The synthesis methods of AgNP are diverse, including physical, chemical, and biological (green) synthesis approaches. Biological synthesis of NPs using bacteria, fungi, algae and plants has emerged as a fascinating alternative to physical and chemical methods due to biocompatibility, self-reducing, capping and stabilizing abilities^9^. The biological synthesis of AgNP is an environmentally friendly and sustainable method, with low cost, low energy consumption and has shown great potential in various fields. Most of the green synthesis is carried out with plant extracts because plants contain many antioxidant molecules (polyphenols, terpenes, favonoids, carboxylic acids, saponins, alcohols and tannins). Plant material, such as leaves, stems, roots, shoots, flowers, barks, seeds and their metabolites have been successfully used^5^.

In addition, bacterial cells are potent “nanofactories” that have been used for the synthesis of various metallic nanoparticles using both intracellular and extracellular routes, although not all bacteria qualify for this purpose. The extracellular route is preferred as there are no downstream processes required for isolation of the final product. Cell-free extracts, bacterial biomass, culture supernatant can be used for extracellular synthesis of nanoparticles. In recent years, the potential of biosynthesis of AgNP using various bacteria has been reported^5,10^. These AgNP have spherical, disk, cube, hexagonal or triangular shapes. The AgNP resulting from each synthetic conditions are unique due to its size, shape, surface area, dispersibility, absorptivity, reactivity and, on top of that, to the unique surface coating. Therefore, using metabolites of specific bacterial strains will result in unique effects due to the bacterial strain metabolites, and enhanced effects due to the small AgNP size. An interesting group of beneficial bacteria are Plant Growth Promoting Bacteria (PGPB), with reported effects to increase plant yield by activating plant metabolism, improving nutrient availability or inhibiting fungal and bacterial growth, preventing therefore disease onset^11,12^. There are previous studies reporting successful AgNP with PGPB, all yielding different type of AgNP^13–18^.

Accordingly, we reasoned that the beneficial strain *Pseudomonas*N5.12, would be an interesting candidate for NPs synthesis if the bacterial metabolites able to trigger plant metabolism and control pathogen growth were present in the coating material, creating a NPs with unique properties. As bacterial and fungal pathogens threaten not only human health but also compromise agronomic yield, this uniqueness makes them an excellent material for agriculture^19^. Hence, the present work explores the ability of bacterial metabolites from the PGPB *Pseudomonas* sp. N5.12 strain to reduce silver, synthetizing AgNP, and their ability to inhibit growth of human pathogenic bacterial and bacterial and fungal phytopathogens. To achieve this objective, the best conditions for biosynthesis, physicochemical characterization and in vitro antibacterial and antifungal activity was evaluated.

## Results

### Biosynthesis and characterization of AgNP

**Fig. 1:**
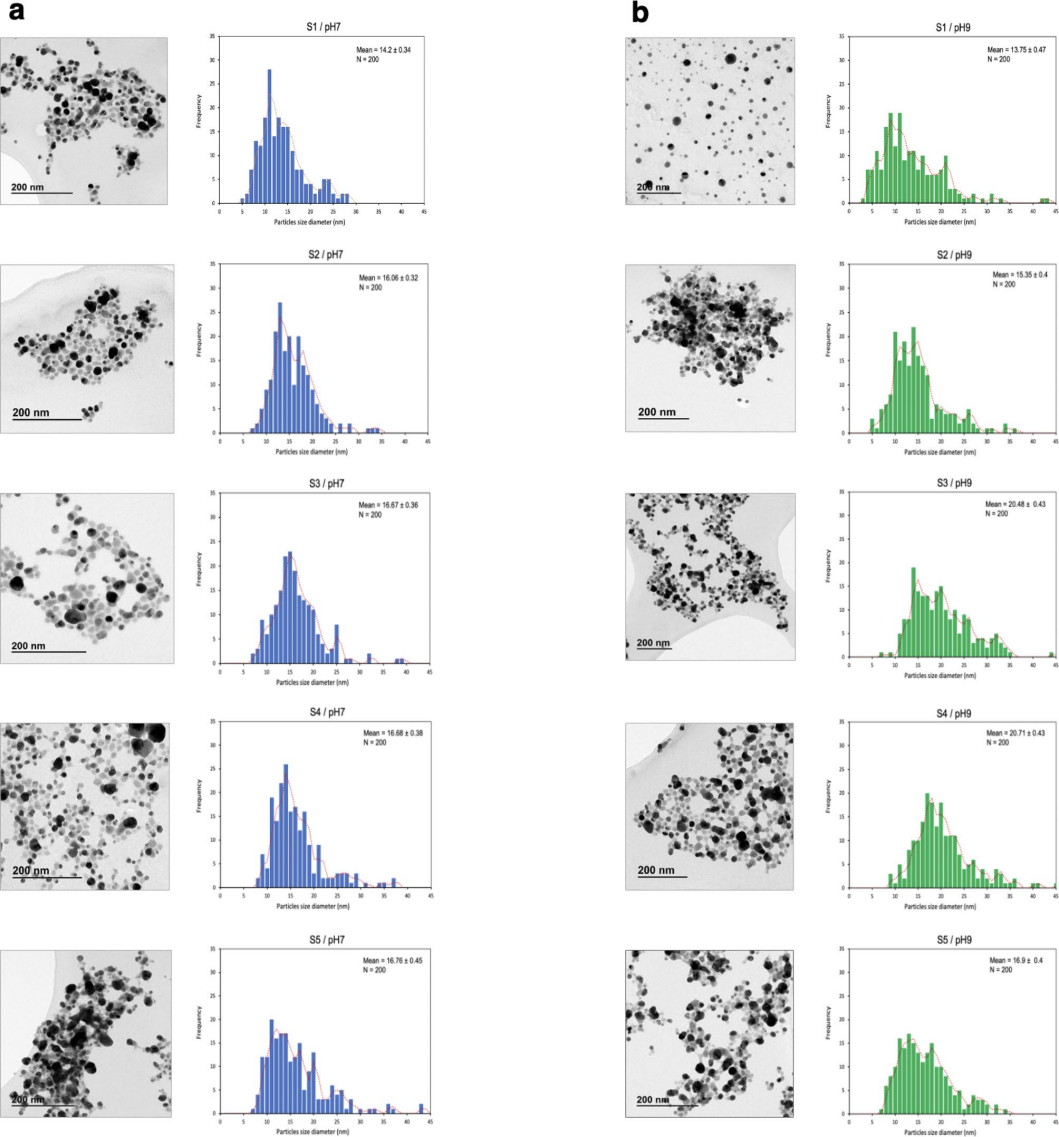
The TEM images and histogram of the particle size distribution of AgNP synthesized at pH 7 **(a)** and pH 9 **(b)**. S1-S5 – different ratio of bacterial supernatant and 1 mM AgNO_3_ solution S1 (5:1), S2 (4:2), S3 (3:3), S4 (2:4), S5 (1:5). The results were recorded as mean ± SD of the total number 200 for each sample. Bar: 200 nm.

AgNP were synthesized by incubation of 1 mM AgNO_3_ with culture supernatant of PGPB *Pseudomonas* sp. N5.12 indicated by the color change of the mixture from light yellow to dark brown; as different volumes of AgNO_3_/supernatant were used, NP are referred to as S1 (1 vol AgNO_3_ /5 vol supernatant) through S5 (5 vol AgNO_3_ /1 vol supernatant). To further confirm the synthesis of AgNP, the UV–vis spectrum was analyzed detecting peaks between 400 and 450 nm, corresponding to the excitation of the surface plasmon resonance of Ag^0^. The maximum wavelength bands corresponding to the different AgNO_3_ /supernatant ratios shifted to larger wavelengths, from 430 to 460 nm. Supernatants filtered after 24–72 h of growing bacteria were also checked for AgNP synthesis resulting in a better performance of the 24-hour supernatant which was selected for further synthesis (Fig. 1 supplementary material). Nucleation reaction temperature greatly influenced the growth of NPs with specific shapes and sizes (Fig. 1 supplementary material) being 37^о^C better for synthesis of AgNP than 28^о^C; therefore, 37^о^C was chosen for further research. The size and morphology of AgNP depends on pH to a great extent, therefore, the effect of pH in the range of 5–9 was studied to optimize parameters for AgNP synthesis. Twenty-four h supernatant at pH5 were not able to reduce Ag^+^ ions as no absorption peak at 420 nm was observed (data not shown). After increasing the pH in neutral and alkaline medium (pH 7 and 9), the absorption peak was in the range 400 to 460 nm, indicating the successful synthesis of AgNP. The size and morphology of AgNP synthesized at pH 7 and 9, with all different AgNO_3_ ratios (S1 to S5) were observed using TEM microscopy (Fig. 1).

The results indicated that the biosynthesized AgNP were mostly a spherical shape and the average size ranged from 14.2 ± 0.34 nm to 16.68 ± 0.38 nm for pH 7, and from 13.75 ± 0.47 nm to 20.71 ± 0.43 nm for pH9. The size, morphology, and distribution of AgNP at different initial concentrations of AgNO_3_ are shown in Fig. 1. Since sample 5 was unstable and precipitated due to the high content of silver nitrate (1:5), it wasn´t used further. Moreover, as the size of the NPs was similar at pH7 and pH9, but the absorption peaks were better in the alkaline medium, samples synthesized at pH9 were selected to continue the study. Two samples (S1 and S4), pH9, with the lowest and highest AgNO_3_ ratios, were used for further characterization.

**Fig. 2:**
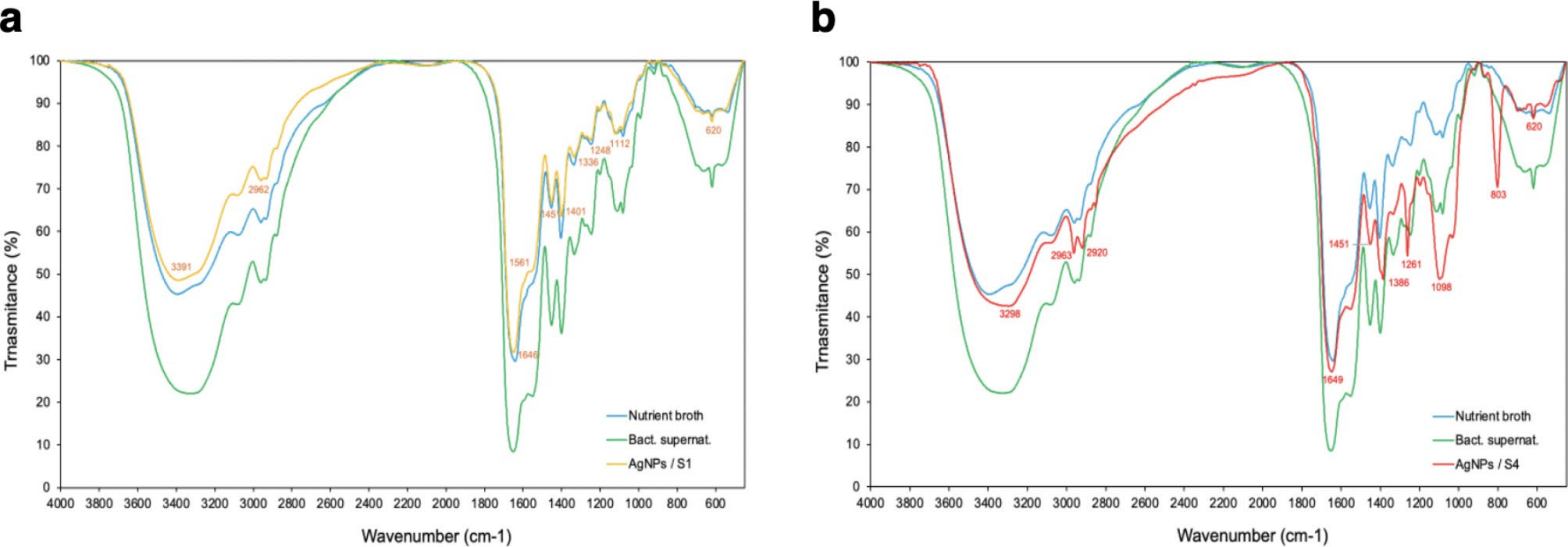
Characterization of components on the surface of AgNP and broths. The nutrient broths and bacterial filtrate before synthesis are shown in green and blue, respectively in both figures; AgNP spectra after synthesis in orange for S1 **(a)** and in red for S4 **(b)**, pH9.

FTIR analyses for identification of functional groups present in the S1 and S4 *Pseudomonas* sp. N5.12 AgNP as well as on raw nutrient broth and supernatant (Fig. 2; Table 1). Overlapping of the 3 elements in each condition revealed different profiles with similar patterns, suggesting a unique coating for AgNP. The main bands of the AgNP spectra would correspond to vibrations of protein bonds (3298–3391 cm^−1^ and 1646–1649 cm^−1^ regions) and carbohydrates (1098–1386 cm^−1^ region), with minor contributions of other biomolecules (https://instanano.com/all/characterization/ftir/ftir-functional-group-search/).

**Table 1:** FTIR analysis of the functional group of NPs for the two studied samples.

AgNP/S1 Visible intensity and wavenumber (cm^−1^)		Vibrational assignment	AgNP/S4 Visible intensity and wavenumber (cm^−1^)	
Wide sharp peak	3391	O–H	3298	Wide sharp peak
Small medium peak	2962	C–H	2920–2963	Small sharp peaks
Very sharp peak	1646	C = O	1649	Very sharp peak
Small sharp peaks	1401–1451	C-H	1451	Medium sharp peaks
Small medium peak	1112–1336	C–O	1098–1386	Very sharp peaks
-	-	C-H	803	Sharp peak
Small sharp peaks	620	N–H	620	Small sharp peaks

**Fig. 3:**
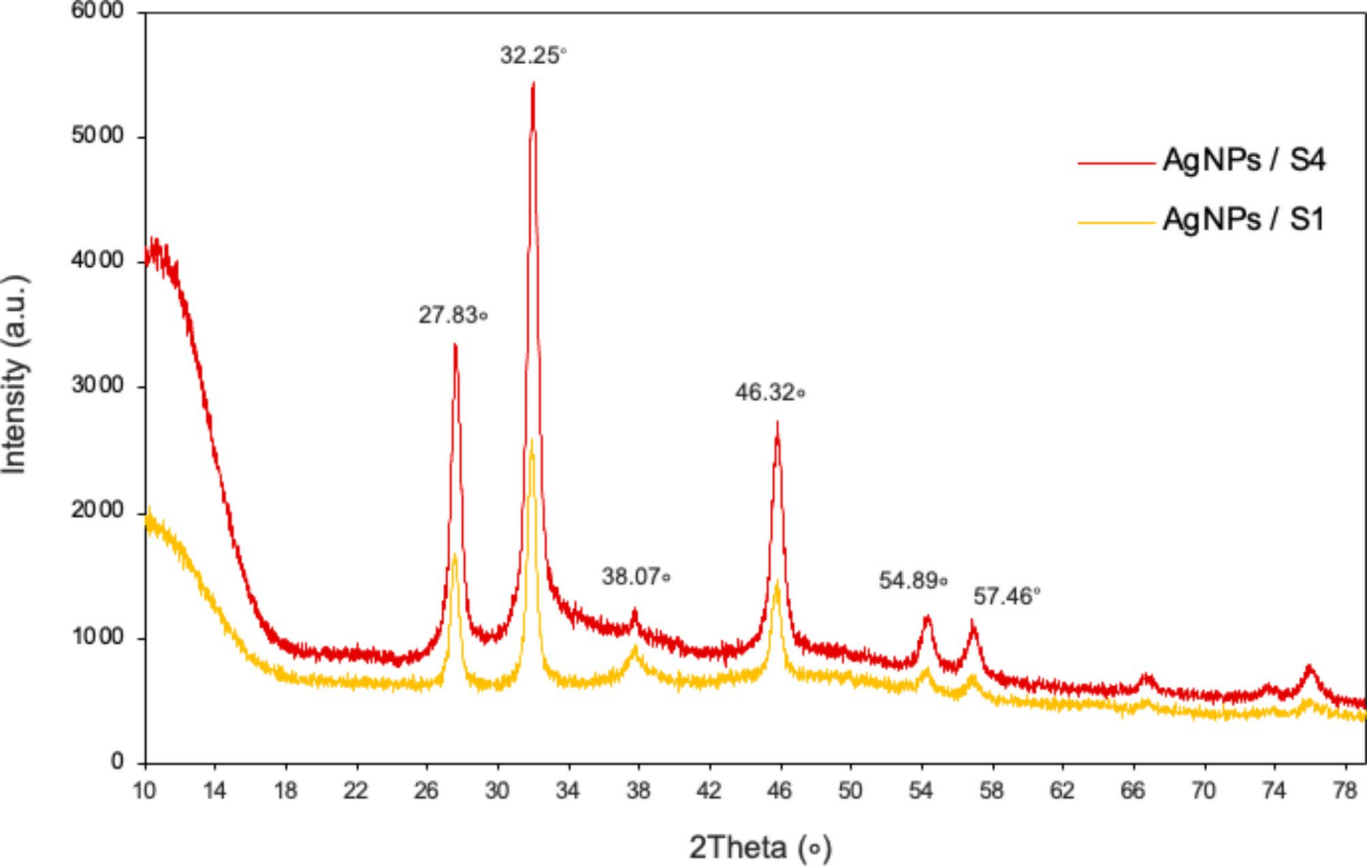
The XRD spectrum of synthesized AgNP (orange for S1 and red for S4, pH9).

The XRD spectrum of the product showed the characteristic Bragg reflection peaks at *2θ* values of 27.83^o^, 32.25^o^, 38.07^o^, 46.32^o^, 54.89^o^ and 57.46^o^. These peaks associated with a face-centered cubic structure of AgCl crystals (Ref. no. 31–1238), thereby confirming the crystalline properties AgNP. Samples 1 and 4 had the same peaks which differed only in the intensity level of these peaks consistent with the higher Ag concentration (Fig. 3).

## Antibacterial and antifungal activity of AgNP

**Fig. 4:**
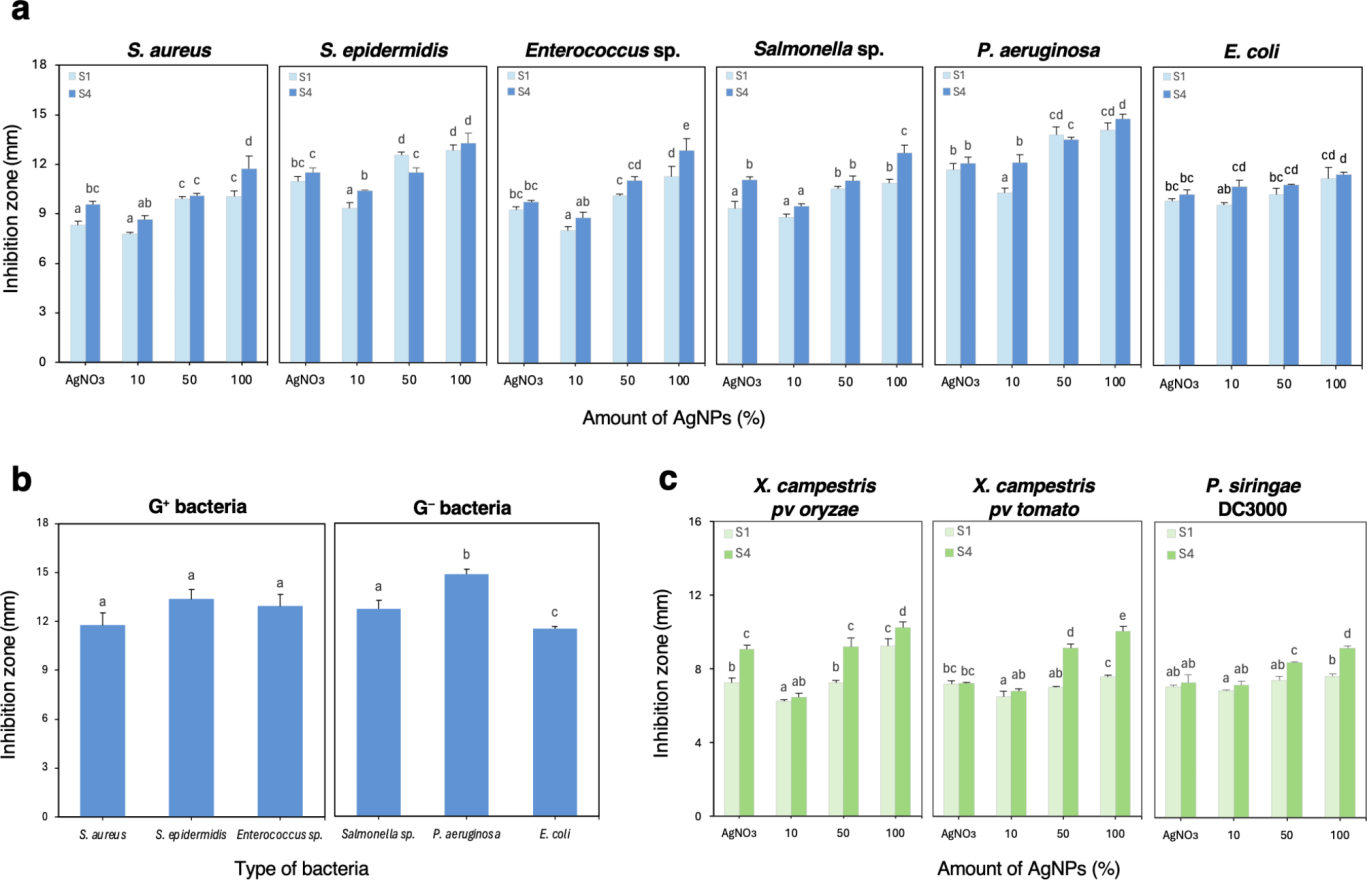
The mean diameter of inhibition zones by AgNP: **(а)** different human pathogenic bacteria; **(b)** different types of bacteria by AgNP/S4 100%; **(c)** plant pathogenic bacteria. The results were recorded as mean ± SD of the triplicate experiment. Different letters indicate significant differences according to LSD (*p* < 0.05).

Two selected AgNP samples (S1 and S4, pH9) were tested for their antimicrobial activity against human pathogenic bacteria such as *Staphylococcus aureus*, *S. epidermidis*, *Enterococcus* sp., *Salmonella* sp., *P. aeruginosa* and *Escherichia coli*. The antibacterial effect was evaluated based on the diameter of the growth inhibition zone at different concentration of NPs and the results are shown in Fig. 4. AgNP were found to have a good inhibitory effect on bacterial growth even when using a 10% NPs solution compared to equivalent 1mM AgNO_3_. The inhibitory effect of the antibiotic standard (gentamicin 10 μm/mL) was significantly higher (*p* < 0.05) than AgNO_3_ controls or bacterial supernatants (Table 2).

Antimicrobial activity was high and concentration-dependent for both samples of NPs in all tested bacterial strains (Fig. 4a); a slightly better effect was observed when using AgNP/S4 (ratio 2:4). Pathogen growth inhibition by AgNP was dependent on the bacterial species; inhibition was always more intense than the equivalent AgNO_3_ concentration, except at the lowest concentration (10%). When using stock concentration (100%), growth inhibition ranged from 13.3 to 21.6% for AgNP/S1, and from 11.6 to 31.7% for AgNP/S4. *Enterococcus* sp. was the most sensitive species in both cases. As S4 was more effective than S1, a comparative analysis of the inhibitory effect of AgNP/S4 at the highest concentration between gram-negative and gram-positive strains was carried out, finding significant differences among the gram-negative only, being *P. aeruginosa* the most sensitive (Fig. 4b). The ability of biosynthesized AgNP to restrict growth of the pathogenic strains has been evidenced.

In addition, we tested our AgNP for their antimicrobial activity against pathogenic phytobacteria (*Xanthomonas campestris pv. oryzae*, *X. campestris pv. tomato* and *P. syringae* DC3000). AgNP also showed inhibitory effect on the tested pathogens, but to a lesser extent than on the human pathogenic strains (Table 2). Again, AgNP/S4 showed better antimicrobial effect than AgNP/S1. Higher concentrations (50%, 100%) significantly inhibited bacterial growth as compared to AgNO_3_ controls (Fig. 4c). *X. campestris pv oryzae* was most sensitive to AgNP/S1 (28.1% less growth) while AgNP/S4 were more effective on *X. campestris pv. tomato*, preventing growth (26.7 to 39.2%) depending on the concentration. These results confirm the dose-dependent antibacterial effect of the studied AgNP. The order of the highest antibacterial activity among plant pathogenic bacteria is *X. campestris pv. oryzae* > *X. campestris pv. tomato* > *P. syringae* DC3000.

**Table 2:**
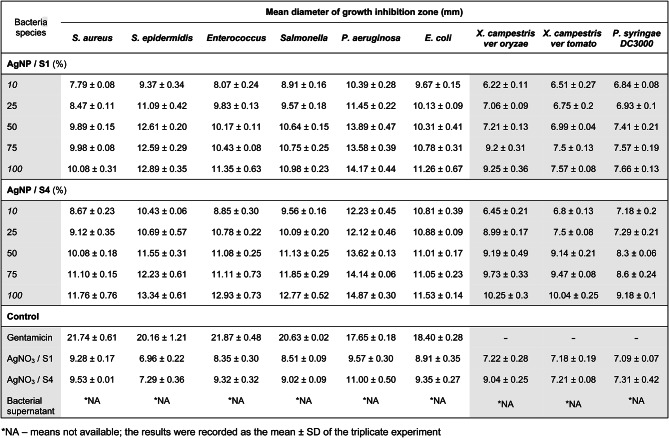
Diameter of the growth inhibition zone of AgNP on pathogens bacteria

**Fig. 5:**
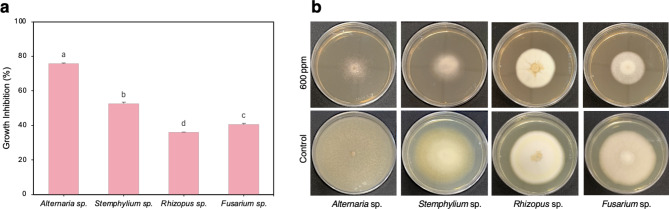
**(a)** Growth inhibition (%) and **(b)** mycelial growth of fungi at 600 ppm AgNP/S4. Results were recorded as mean ± SD of a triplicate experiment. Different letters indicate significant differences according to ANOVA and LSD post hoc test (*p* < 0.05).

The antifungal activity of AgNP was evaluated in triplicate on four common phytopathogenic strains, as *Alternaria* sp., *Stemphylium* sp., *Rhizopus* sp. and *Fusarium* sp. The results showed that the inhibition of the mycelial growth caused by AgNP varies depending on the fungal species, concentration and type of NPs (S1 or S4) (Table 3). At 60 ppm, all fungi tested achieved less than 10% inhibition. At high concentrations (600 ppm) AgNP/S1 showed good inhibitory activity, being most effective on *Alternaria (*41.4 ± 1.6%) *sp*. AgNP/S4 were more efficient (Fig. 5). With the highest dose (600 ppm), the most sensitive was *Alternaria* sp., (75.7 ± 0.56% growth inhibition), followed by *Stemphylium* sp. (52.57 ± 0.95%), *Fusarium* sp. (40.52% ± 0.65) and *Rhizopus* sp. (35.86% ± 0.30).

**Table 3:** Growth inhibition (%) of phytopathogenic fungi by AgNP/S1 and AgNP/S4 relative to pathogen controls.

Fungi strain	Mycelial Growth Inhibition (%)			
	AgNP/S1		AgNP/S4	
	60 ppm	600 ppm	60 ppm	600 ppm
*Alternaria* sp.	2.33 ± 0.15	41.37 ± 1.58	8.16 ± 0.59	75.70 ± 0.56
*Stemphylium* sp.	4.11 ± 0.84	19.57 ± 3.19	9.97 ± 2.92	52.58 ± 0.95
*Rhizopus* sp.	5.59 ± 0.22	20.84 ± 0.37	2.61 ± 0.69	35.86 ± 0.30
*Fusarium* sp.	6.30 ± 2.88	25.94 ± 1.02	3.48 ± 1.30	40.52 ± 0.65

## Discussion

The biocontrol capacity of certain Plant growth promoting rhizobacteria of the *Pseudomonas *genera has been reported before and is usually associated to a plethora of secondary metabolites, siderophores or lipopeptides^20^. In this work, we used the metabolites of the siderophore-producing plant growth-stimulating *Pseudomonas *sp. N5.12 for biological synthesis of AgNP, determining the best conditions for synthesis, and explored the NP´s ability to control bacterial and fungal growth. Biological synthesis of AgNP has gained popularity as a potential alternative to other chemical and physical methods, being plant extracts preferred for their high antioxidant potential. However, microorganisms can accumulate and detoxify heavy metals due to the presence of various cytoplasmic reductase enzymes as well as various metabolites released by bacteria, being both effective in the formation of AgNP, creating unique NP depending on metabolites^21^. In recent research, bacteria such as *P. aeruginosa*,* P. canadensis*, *Bacillus zanthoxyli* GBE11, *B. subtilis* and *E. coli *have been shown to be able to synthesize AgNP^22–25^. In this work, we used the plant growth-stimulating bacteria *Pseudomonas *sp. N5.12 for biological synthesis of AgNP, which exhibited a yellowish-brown color from the excitation of surface plasmon vibrations of metal NPs in all evaluated conditions. Surface plasmon vibrations are caused by dipole oscillations which arise as a result of the combination of electromagnetic field oscillations in the visible range with collective oscillations of conduction electrons^26^. The absorbance peak at 400–450 nm in the UV-visible spectrum corresponded to the typical band of AgNP generated by the ability of bacterial metabolites to reduce Ag^+^ [^13,23^]. According to TEM data (Fig. 1), the biosynthesized AgNP had a spherical form with a size range of 13.8 to 20.7 nm being the differences in nanoparticle size due to pH and AgNO_3_concentration^13,14,23^. Setting the variables for nucleation (temperature, incubation time and pH) is key for the final properties of the AgNP, with reported nucleation time ranges from 24 to 200 h, temperature ranges from 25 to 50 °C, and pH 4–9 when using bacterial filtrates from other *Pseudomonas* sp (Table 4). Regarding which of these factors would be more relevant in the process, based on our experience, pH would be of primary influence as it would be determining the charge of specific organic groups present in bacterial metabolites capable of reducing Ag^+^ as shown by preliminary data showing that pH5 results in total failure of the process, while pH7 and pH9 contribute to successful synthesis. Nevertheless, presence of presence of bacterial enzymes among these metabolites cannot be ruled out either, and all evaluated factors determine enzyme performance potentially involved in Ag^+^reduction. The importance of synthesis conditions may vary depending on the biological material used for NP synthesis. Different matrices may require different initial conditions to achieve optimal biological NP formation^1,2^. The effectiveness of these conditions can vary not only between species but also within different strains. Therefore, while pH is effective for *Pseudomonas* N5.12, other factors, such as temperature, time, or metal concentration, may be crucial for nanoparticle synthesis for another biological sources. Therefore, setting conditions for the synthesis are very important, and the resulting AgNP will differ in their characteristics, even if they are synthesized from the same genus of bacteria (Table 4, and references therein).

**Table 4:** Biological synthesis of AgNP using various *Pseudomonas* sp.

Strain	Time cultivation	Used materials	Concentra-tion AgNOз	Synthesis parameters	Wavelength UV-Vis	Size (nm) Shape	Ref.
*Pseudomonas* sp. THG-LS1.4	24 h, 28 ° C 120 rpm	Supernatant	1 mM	48 h, 28 ° C	412 nm	10–40 nm	^14^
*P. rhodesiae* G1	24 h, 30 ° C 200 rpm	Supernatant	1 mM	48 h, 30 ° C 200 rpm	420–430 nm	20–100 nm Spherical	^13^
*P. fluorescens* YPS3	24 h, 37 ° C	Supernatant	1 mM	1 h, dark conditions	420 nm	13–54 nm Spherical	^15^
*P. fluorescens*	72 h, 28 ° C 120 rpm	Biomass	1 mM	24 h, room temperature	420 nm	30 nm Spherical	^16^
*P. fluorescens*	-	Supernatant	0.1 mM	2 h, 80 ° C pH 5	-	10–100 nm Spherical	^27^
*P. deceptionen-sis* DC5	24 h, 37 ° C 120 rpm	Supernatant	1 mM	48 h, 25 ° C 200 rpm	428 nm	10–30 nm	^28^
*P. canadensis*	24 h, 28 ° C 150 rpm	Biomass	0.5–4 mM	48–72 h, pH 5–9 20–35 ° C	425 nm	21–52 nm Spherical	^23^
*P. alloputida* B003 UAM	30 ° C 150 rpm	Supernatant	1 mM	40–80 h 180–200 h	410–411 nm 404–420 nm	31–35, 20–24 nm Spherical	^29^
*P. aeruginosa* KUPSB12	24 h, 37 ° C 200 rpm	Biomass	1 mM	48 h, dark room conditions	442 nm	50–85 nm Spherical	^30^
*P. aeruginosa* BS-161R	72 h, 35 ° C 150 rpm	Supernatant	1 mM	1–8 h, 28 ° C 200 rpm	430 nm	8–24 nm Spherical	^31^
*P. aeruginosa*	24 h, 37 ° C 150 rpm	Supernatant	1–4 mM	48 h, pH 4–8 28–50 ° C	410–420 nm	11–25 nm Square/ aspherical	^17^
*P. aeruginosa*	48 h, 27 ° C 200 rpm	Supernatant	1 mM	1–24 h, 7–15 d 27 ° C	420 nm	30–75 nm Spherical	^18^
*P. aeruginosa*	72 h, 37 ° C 150 rpm	Supernatant	0.1 g/L	72 h, bright conditions	420–430 nm	20–100 nm Spherical	^32^

In addition to size and shape, the biomolecules responsible for the capping and stabilizing of nanoparticles make NP unique. According to FTIR analysis, we confirmed that AgNP are surrounded by a corona of organic compounds. The main bands of the AgNP spectra would correspond to vibrations of protein bonds (3298–3391 cm^−1^ and 1646–1649 cm^−1^ regions) and carbohydrates (1098–1386 cm^−1^ region), with minor contributions of other biomolecules; a band was observed at 803 cm^−1^ unique to S4 AgNP (Fig. 2). The free amine and carbonyl groups present in the bacterial protein could possibly perform the function for the formation and stabilization of AgNP. FTIR studies of other synthesized green AgNP have reported different types of compounds, but in most cases proteins and lipids are found to be present^13,29^. These results suggest that, although different conditions of synthesis are used (S1 and S4), AgNP tend to be surrounded by the same kind of organic compounds (Table 1). Probably, differences in corona components exist, in terms of the proportion of each type of a particular compound, producing properties that may diversify its functionality. The crystalline nature of silver was confirmed by XDR analysis showing five diffractions peaks that correspond to the face-centered cubic structure of AgCl crystals. Previous research that demonstrated the creation of AgNP utilizing microbes had comparable XRD results^29,33^. The detection of AgCl on XRD is explained by the presence of NaCl in the broths used in the synthesis of NPs.

Prior to evaluation of antibacterial potential, the number of different AgNP obtained was narrowed down to 2 types, assuming from the beginning that the bacterial metabolites were the key to this process. We were looking for the best NP, based on (i) size, to ensure effectiveness, and (ii) coated with metabolites, to provide a new interesting approach to NP. As metabolites were always present in all conditions, we selected pH9 over pH7 for intensity of reaction (UV-spectrum), and among those, we chose the two NP most different in size and Ag content to test antimicrobial potential, assuming that intermediate condition would not represent a big difference.

AgNP are the most investigated antibacterial agents against various bacterial and fungal pathogens, both relevant for human and plant health^34^. The true relevance of this enhanced antibacterial potential of AgNP relies on the expectation to control growth of multi drug resistant strains, which limit the ability to control disease spread. When talking about agriculture, bacterial pathogens account for 16% yield losses, while fungal pathogens are responsible for up to 80% loss in yield^35^, compromising food availability, despite the many chemicals used for this purpose. Hence, finding effective alternatives to cope with this challenge is of paramount importance.

The present study showed that all the studied human pathogenic strains were sensitive to N5.12 unique AgNP, being Gram-negative bacteria strains more sensitive. Consistent with previous reports the stronger inhibitory effect on Gram-negative is due to differences in cell wall composition^36,37^. Moreover, our NPs also showed antibacterial activity against phytopathogenic bacteria, especially relevant in S4 AgNP (Fig. 4c; Table 2). The reported mechanisms of action to inhibit bacterial growth consist in interaction with the outer bacterial membrane. AgNP easily adhere to the cell wall and cytoplasmic membrane due to electrostatic attraction and their accumulation on surface of membrane. As a result, the permeability of the bacterial membrane is disturbed which leads to the destruction of cells^38^. The intracellular action of silver ions consists in the deactivation of the respiratory enzymes and generation reactive oxygen species which leads to DNA modification. AgNP interact with sulfur and phosphorus in DNA which can cause damage to DNA replication and cell death^39^. However, these mechanisms necessary imply that the AgNP behaves as a source of Ag ions, which is determined to an unknown extent by the capping agents that conform the corona^40^. According to our results (Fig. 4; Table 2), and inhibition was higher than AgNO_3_ equivalent concentration, hence speaking of an effective corona composition able to biocontrol bacterial phytopathogens, other than the ion-effects. Hence, the exact mechanism by which N5.12 AgNP control bacterial and fungal growth is probably based in specific bacterial active molecules present in the corona and is yet to be determined.

Interestingly, NP controlled *Stemphylium* growth by 52.6% (Fig. 5; Table 2); considering that this fungi compromises crop yield i.e. onion yield by 90%^41^, lentil by 100%^42^or sugar beet^43^ among others, this result is a very interesting active matter for agriculture. Similarly, this may benefit other crops which are affected by *Alternaria *sp., which is also controlled even to a greater extent (75%)^44^. The least efficiency of AgNP was observed against *F. oxysporum* (68%) inhibition of colony formation. Despite of the minor inhibition on *Fusarium*, N5.12 AgNP would be an excellent tool when no other products are available. Thus, AgNP exhibited strong antifungal effects on tested fungi and biosynthesized AgNP can be applied effectively in the control of phytopathogens and the prevention of deleterious infections.

In view of the inhibitory potential of N5.12 AgNP, and before developing a product for agriculture, it is convenient to raise concern about the potential risk for the environment. Further experimental work is necessary to evaluate the potential risk for agriculture. However, as the corona is made of metabolites from beneficial rhizobacterial strains, a low risk for the environment is anticipated.

AgNPs are proposed as antimicrobial agents in agriculture but evaluating their cost-effectiveness and feasibility for large-scale application must be addressed. AgNPs must also be durable and effective in diverse agricultural environments to be cost-efficient. Potential barriers to widespread adoption include environmental and regulatory concerns, particularly regarding soil health and microbial communities. Public perception and competition from traditional pesticides or organic alternatives may also hinder their use. Additionally, the infrastructure required for large-scale production, distribution, and application adds complexity and cost. In summary, while AgNPs show promise, their large-scale adoption will depend on overcoming production challenges, regulatory approval, and integration into existing agricultural practices. On the other hand, and looking into circular economy, this technology represents an advantage to industries devoted to production of biostimulants and biofertilizants. These industries produce high volumes of fermentation by-products as they only use bacterial cell for marketable microbial products. Once cells are removed, metabolites need to be eliminated. Using them for NP is a process to increase company value, creating innovative products, probably next-generation antimicrobials.

In summary, bacterial metabolites of the PGPB *Pseudomonas* sp. N5.12 have been able to reduce Ag^+^, resulting in biological synthesis of AgNP, for which the best synthesis conditions were determined. Characterization of the AgNP revealed 13 to 20 nm spherical AgNP, with a unique composition of the organic corona, which is related to their antibacterial activity against human and plant pathogens. Based on the obtained results of antibacterial activity, the potential use of these AgNP as a means of control against human pathogens is possible, but its applications for agriculture foresee great success based on antifungal activity.

## Materials and methods

**Preparation of *Pseudomonas*sp. N5.12 strain culture**.

*Pseudomonas sp. N5.12* was isolated from the rhizosphere of *Nicotiana glauca*in Almeria, Spain^45^. The bacterial strain was periodically cultured and maintained in plate count agar (PCA). For biosynthesis of AgNP, *Pseudomonas* sp. was grown in nutrient broth (NB, Pronadisa Spain) at 28 °C in an orbital shaker, at 150 rpm, for 24, 48 and 72 h. The bacterial culture was centrifuged at 5000 rpm for 20 min using a refrigerated centrifuge (AFI LISA MultiLab Centrifuge, France). The pellet was discarded, and the supernatant was filtered by 0.25 mm (VWR International, USA) and used for the extracellular synthesis AgNP. Cell-free culture supernatant of *Pseudomonas* sp. N5.12 was confirmed by no bacterial growth after incubating 100 µL of supernatants on nutrient agar at 28 °C for 24 h.

## Biosynthesis of AgNP

The following variables were considered to determine the optimal conditions for NPs biosynthesis: pH (5, 7, 9), ratio supernatant/AgNO_3_ (v: v, (5:1, 4:2, 3:3, 2:4 and 1:5), and nucleation temperature (28–37ºC). The culture supernatants were adjusted to corresponding pH. Then, supernatants were mixed with 1 mM AgNOз aqueous solution in different ratios. Flasks were incubated for 24 h orbital shaker at 150 rpm under continuous light conditions; nutrient broth with AgNOз solution used as control, to rule out presence of reducing metabolites as medium components. After incubation period, the AgNP were collected by centrifugation at 5000 rpm for 20 min (AFI LISA MultiLab Centrifuge, France) and washed thoroughly with miliQ water to remove the unconverted metal ions or any other constituents, stopping nucleation. Presence of NPs was confirmed by UV absorption as described below. The purified AgNP were freeze-dried and liophylized to obtain a powder. A stock solution dissolved in 1 mL distilled water was prepared for further characterization and application in antimicrobial activity experiments. The procedure for the biosynthesis of AgNPs, with all conditions outlined, is presented in Fig. 2S (supplementary material).

### Characterization of synthesized AgNP

The bioreduction of the Ag^+^ ions in the solution were observed by changes in color from light yellow to dark brown. The absorption spectrum of this solution was monitored by UV–Visible spectrometry from 200 to 800 nm at 1 nm resolution using a SPECTROstar Nano spectrometer (BMG LABTECH, Germany) confirming presence of Ag^0^ by increased absorption at 420–450 nm.

Size distribution and morphology of synthesized AgNP were estimated by TEM. The equipment is Thermo Fisher Scientific Prisma E and xT Microscope Control v16.2.2 software. The measurements have been carried out by depositing a few drops of the sample in suspension on aluminum and magnesium sample holders, allowing it to dry before placing it in the microscopes. In the black and white images, topography, we have worked with the Everhart Thornley Detector (ETD) of secondary electrons at 30 kV and spot 2.0 (= 24 pA). Analyses were carried out at ICTS-CNME (https://cnme.es/).

Functional groups responsible for the synthesis and stabilization of AgNP were detected by Fourier transform infrared (FTIR) spectroscopy, on KBr pellets. The samples were scanned using a Spectrum Two FTIR Spectrometers (Perkin Elmer) with a resolution of 4 cm^−1^ and a range of 450–4000 cm^−1^. Analyses were carried out at SIDI (https://www.uam.es/uam/en/sidi/unidades-de-analisis/unidad-analisis-estructural-molecular/ftir).

The lyophilized samples of the biosynthesized AgNP coated on XRD grids were studied for X-ray diffraction patterns using a Bruker D8 diffractometer and a fast detector LynxEye, operating at a voltage of 40 kV and current of 40 mA with a scan rate of 0.01/s (https://www.uspceu.com/investigacion/servicios-apoyo/servicio/difracci%C3%B3n-de-rayos-x).

## Antibacterial activity of AgNP

The antibacterial disk diffusion assay on antibacterial activity of biosynthesized AgNP was evaluated using the Kirby-Bauer technique^46^. Three Gram-negative (*Salmonella* sp., *Escherichia coli* and *Pseudomonas aeruginosa*) and three Gram-positive (*Enterococcus* sp., *Staphylococcus epidermidis and Staphylococcus aureus*) human pathogen bacterial strains were used in this study. Also, three phytobacteria were tested in this study: *Xanthomonas campestris pv. oryzae*,* X. campestris pv. tomato* and *P. syringae* DC3000.

Mueller Hinton Agar (MHA) was used for the analysis of antibacterial activity on disposable sterile Petri dishes and stored in the refrigerator at 4 °C for further use. Gentamicin (10 µg/mL), AgNO_3_ 1mM solution in equivalent concentrations to NP, and cell-free culture supernatant of *Pseudomonas* sp. N5.12 were used as positive and negative controls. The bacterial pathogen suspension was prepared by subculturing bacteria into NB medium. The human pathogenic bacteria were incubated for 24 h in orbital shaker at 150 rpm for 37 °C; phytopathogenic bacteria were incubated at 28ºC. The optical density OD600 of the bacterial suspension was adjusted to 0.1 absorbance at 600 nm, that corresponds to 1.5 × 10^6^ cfu/mL. The inoculum with bacteria culture (100 µL) was then spread evenly over the MHA plate using a sterile disposable spatula. Paper discs (6 mm) containing 30 µL of the AgNP at different concentrations (10, 25, 50, 75 and 100%) were placed on the center of the petri dishes. The diameter of the growth of each bacteria was measured by digital image analysis using ImageJ software (Version 1.8.0_345). The results were recorded as the mean ± standard deviation of the triplicate experiment. The experiment was carried out in triplicate and measurements were made after 24 h of incubation at 37 °C for human pathogens while phytopathogens were incubated at 28ºC.

## Antifungal activity of AgNP

The effect of AgNP was studied on *Fusarium* sp., *Stemphylium* sp., *Alternaria* sp. and *Rhizopus* sp. fungi. The Potato-Dextrose-Agar (PDA) culture medium was prepared, sterilized and stored in the refrigerator at 4 °C for further use. AgNP were added to the medium at concentrations of 60 and 600 ppm. A PDA disk with fresh fungal mycelium of 0.5 cm in diameter, was placed in the center of each Petri dish with NPs in the medium; plates were incubated at room temperature, in the dark, until mycelial growth in controls covered the surface, variable according to each fungus. The diameter of the growth of each fungus was measured by digital image analysis using ImageJ software (Version 1.8.0_345). The mycelial growth was measured in every Petri dish after 5 days after incubation for *Alternaria* and *Stemphylium* spp. and 12 days after incubation for *Fusarium* and *Rhizopus *spp. The percentage of mycelial growth inhibition was calculated using the following equation proposed by^47^: Inhibition% = (DCC − DCP)/DCC × 100%, where: DCC – diameter of the control colony (cm); DCP – diameter of the problem colony (fungus in the presence of AgNP) (cm). Each treatment had three replicates.

### Statistical analysis

All antimicrobial experiments were conducted in three independent replicated and the results were stated as the mean ± standard deviation (mean ± SD) analyzing by one-way analysis of variance (ANOVA) between the obtained values (*p* < 0.05) and LSD post hoc test. The statistical data analysis was analyzed using IBM SPSS Statistics (Version 29.0.2.0). Values of *p* < 0.05 indicated the significant differences.

## Electronic supplementary material

Below is the link to the electronic supplementary material.

Supplementary Material 1

## Data Availability

The datasets generated and/or analysed during the current study are available in the Zenodo repository, https://doi.org/10.5281/zenodo.14051744.
