# Supplementary material for: Silver nanoparticles coated with metabolites of Pseudomonas sp. N5.12 inhibit bacterial pathogens and fungal phytopathogens
Source: Sci Rep. 2025 Jan 9;15:1522. doi: 10.1038/s41598-024-84503-z (PMC11717911; doi:10.1038/s41598-024-84503-z)
Supplement: Supplementary file 1 — Supplementary Material 1 [file 41598_2024_84503_MOESM1_ESM.docx]

**Supplementary materials**


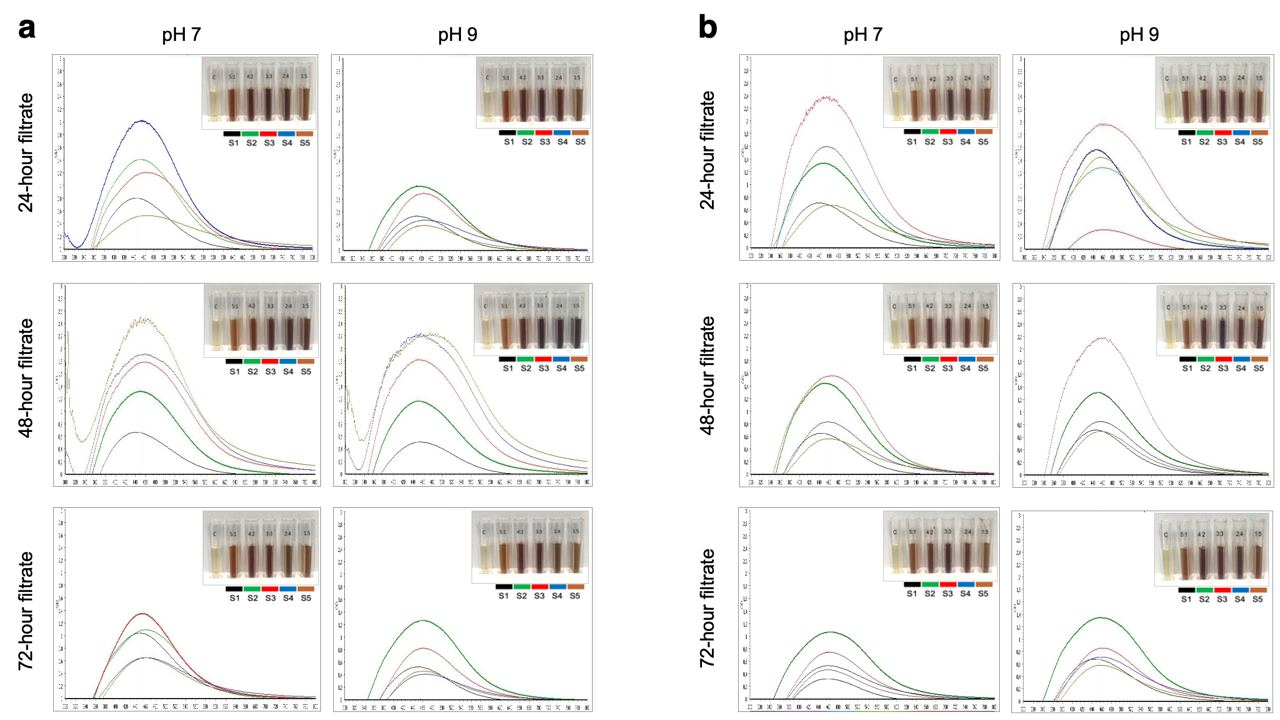


**Figure 1Supp.** The UV-vis spectra of AgNP synthesized under different conditions at temperature of 28^о^C **(a)** and 37^о^C **(b)**. S1-S5 – with bacterial metabolites (supernatants) produced after 24, 48 and 72h, using different ratio of bacterial supernatant and 1 mM AgNO_3_ solution (v/v) (5:1, 4:2, 3:3, 2:4, 1:5).


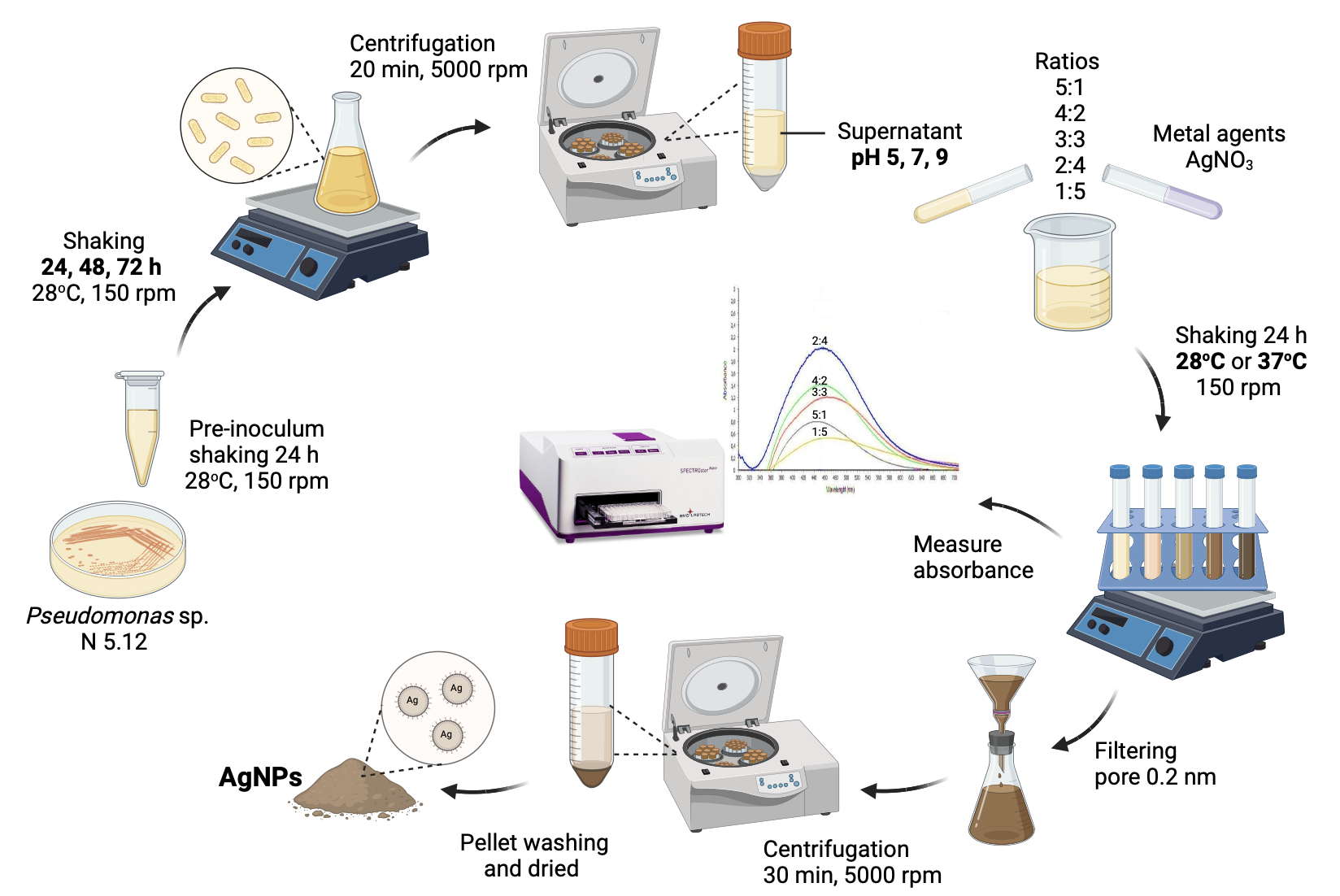


**Figure 2Supp.** Scheme of biosynthesis of AgNPs from *Pseudomonas* sp. N5.12​ under different conditions.
